# The Right of the Child

**Published:** 1921-02-19

**Authors:** 


### THE PUBLIC WELL-BEING.

### THE RIGHT OF THE CHILD.

### AN APPEAL TO THE MODERN MOTHER.

In the *International Journal of Public Health*, Professor Francesco Valagussa, discussing post-war conditions in the feeding of children, speaks out with trenchant force and with admirable candour on the subject of the nursing mother. Milk, he says, is not to be regarded as a solution of carbohydrates and salts, a colloidal suspension of albuminous substances, or an emulsion of fats. It is a physiological fluid, a living fluid, into which go all the specific, vital elements of the species to which it belongs, and which cannot with safety take the place of another or be replaced by another. That sentence furnishes the text of a powerful, but not exaggerated sermon--expressed in simple language—which might be read without difficulty and with advantage by every modern mother.

If it is always the duty of the mother, except in certain well-defined cases of illness, to nurse her own offspring this duty is especially imperative in time of want in order to assure physiological development of the new-born child. Maternal nursing may be said to supply the "nectar of life." From it are derived the elements of future health and strength, the means of defence and the harmonious action of the various organs and systems of the body. From a summary of many statistics concerning mortality in relation to the methods of feeding, it is shown by this Italian doctor that thirteen per cent. of babies die when maternally nursed under well or badly regulated conditions; thirty-three per cent. when breast-fed at home by a wet-nurse; fifty per cent. when nursed by a supervised person away from home; and sixty per cent. when the nurse is not supervised. To-day, more than ever must it be said to the mother, "Nurse your children if you wish to see sound and healthy men prepared to give their country reconstructive energy after so many destructive calamities." It is not that the mothers have less milk through any inherited atrophy from disease of the nursing function. Our women, declares the author, have the breasts to nurse their children; it is only the will and the desire to do so which are lacking in some members of the well-to-do classes who fear that nursing will limit their liberty.

### A Good French Law.

It is true to say that the new-born child has an absolute right to its own mother's milk. While in Great Britain, at any rate, the practice of breast-feeding by a wet-nurse has very greatly diminished in recent years, there is something to be said for the application in every country of such a law as that of Roussel, which has been in force for many years in France, and which forbids the peasant women to hire themselves out as nurses for the children of the wealthy before having nursed their own children for a certain time. "We who see at close quarters," comments this very plain-spoken writer, "the misfortunes of infancy consider it inhuman and often criminal, for the nursing mother to abandon her baby through the desire for gain, entrusting her new-born child to some neighbour who may be syphilitic, or without sufficient milk, and who may feed the poor infant on some gruel or bean soup instead of nature's food. These wretched babies are wrinkled and thin, with huge abdomens and deformed bones, their skins covered with crusted dirt; while the children of the wealthy, who thus take mother's milk which does not belong to them, grow, thrive, and fatten at the expense of their foster brothers." That is a true picture of a practice which is essentially immoral because it is contrary to nature, and therefore inimical to the balance of life. If the desire for gain dulls the maternal instinct common to every animal species, the State may be compelled to make laws for protecting the offspring of the poor as well as the wealthy. The value of the life of the new-born child is identical in all cases, and the State must judge between sentimental considerations, instincts, and social classes.

In cities no wet-nurses, in Colonel Valagussa's opinion, ought to be obtainable who have not been lactating for at least six months. This would certainly have the advantage of safeguarding through the most difficult period of nutrition the life of the peasant's child, the life of the child of the people, and might diminish the infant mortality rate nearly by half. It would also oblige many wealthy mothers, far too ignorant of the true meaning of maternity, to nurse their infants and realise the importance of fulfiling their highest responsibilities. Another factor, more prominently recognised of late, requiring that mothers should nurse their own children is the scarcity both in quality and numbers of wet-nurses. The war, if it has not lessened the number of births, has probably lessened the number of nurses whose health one can be sure of; absence of husbands called to the army has increased the number of syphilitic infections, and therefore the dangers from the syphilitic wet-nurses have been multiplied.

### The Cost of State Endowment.

It is not surprising, in view of his general attitude towards this subject, to find the author of the article advocating that it is the duty of society to guarantee the nursing mother's existence "as a fundamental element of present and future life." In plain words what he seeks to bring about is a State endowment of motherhood in all civilised nations. That is an ideal which strongly appeals to one class of philanthropists, but to another class

**THE PUBLIC WELL-BEING**—(*continued*).

suggests the indirect danger of removing from parents the sense of immediate responsibility and self-respect. In any case it seems for the present to be outside practical politics at least so far as Great Britain is concerned. There are so many more urgent health problems demanding to be solved, and in view of the heavy national indebtedness the expenditure, proposed and actual, of the Health Ministry is being regarded with such jealous scrutiny by a public calling out for economy on every hand, that no wide scheme for the endowment of motherhood could be seriously entertained. It was recently estimated that a scheme of this kind on the broadest basis would cost the country the vast sum of 240 millions per annum, and if narrowed down to the minimum application would still cost forty millions a year. Much useful service can yet be done, both in education and advice and in practical assistance, by voluntary institutions favouring maternal nursing and at the "welcome" centres established in all parts of the country. And the teaching on this subject to be given to all mothers can be summarised in a sentence: In well-regulated breast-feeding lies the secret of the child's vigour and vitality, his ability, when weaned, to tolerate other foods, his freedom from minor ailments and his resistance to disease.

